# THE EFFECT OF GERMANY’S LONG-TERM CARE STRENGTHENING ACT II ON NURSING HOME USE

**DOI:** 10.1093/geroni/igad104.0123

**Published:** 2023-12-21

**Authors:** Freya Diederich

**Affiliations:** University of Bremen, Bremen, Bremen, Germany

## Abstract

In Germany, out-of-pocket costs for nursing home care have substantially increased during the last years. There is an ongoing discussion whether these out-of-pocket costs should be capped. However, a decrease in costs may increase nursing home use and the social costs of nursing home care. This study analyzes how a change in out-of-pocket costs for nursing home care affects nursing home use. We use discontinuities in out-of-pocket costs that arose from Germany’s Long-Term Care Strengthening Act II in 2017. The Long-Term Care Strengthening Act II decreased average nursing home care costs for people with higher care needs and increased average costs for people with lower care needs. Long-term care insurance data are analyzed using a regression discontinuity design. There is no significant change in nursing home use among people who enter long-term care with relatively high care needs. However, there is a significant decrease in nursing home use among those who enter long-term care with relatively low care needs. The results are robust regarding different specifications of the model. We conclude that the elasticity of nursing home use depends on the level of care needs. For people with high care needs, nursing home care is often without alternatives, whereas people with lower care needs may substitute nursing home care with informal care. A generous public policy does not necessarily increase demand for nursing home care.

